# Supplementary material for: School closures significantly reduced arrests of black and latinx urban youth
Source: PLoS One. 2023 Jul 26;18(7):e0287701. doi: 10.1371/journal.pone.0287701 (PMC10370768; doi:10.1371/journal.pone.0287701)
Supplement: S1 Table — (DOCX) [file pone.0287701.s001.docx]

**S1 Table.** Negative binomial regression results of arrests by age group before and after school closures, 2019-2020

|  | Youth  (<18) | Young adults  (18-24) | Adults  (25-64) | Older adults  (65+) |
| --- | --- | --- | --- | --- |
| Remote period | -.777*** | -.493*** | -.551*** | -.735*** |
|  | (.058) | (.038) | (.030) | (.059) |
| Partial return | -.747*** | -.118 | -.257*** | -.356*** |
|  | (.090) | (.069) | (.052) | (.074) |
| Charleston | 1.618*** | 1.615*** | 1.277*** | 1.557*** |
|  | (.094) | .(052) | (.032) | (.141) |
| New York City | .732*** | 1.630*** | 1.066*** | 1.075*** |
|  | (.069) | (.041) | (.028) | (.096) |
| Pittsburgh | 1.476*** | 1.334*** | 1.514*** | 1.738*** |
|  | (.086) | (.044) | (.032) | (.118) |
| Intercept | -9.810*** | -8.430*** | -8.467*** | -11.235*** |
|  | (.102) | (.044) | (.032) | (.110) |
| *N* city-weeks | 418 | 418 | 418 | 418 |

*p<.0001*** p<.001** p<.0.01**

*Note*: Robust standard errors in parentheses. Month fixed effects are suppressed. Reference for time period is 2019-March 2022. Reference category for city is Boston. Main results reported as IRR in Fig 2 of main paper.
