# Supplementary material for: School closures significantly reduced arrests of black and latinx urban youth
Source: PLoS One. 2023 Jul 26;18(7):e0287701. doi: 10.1371/journal.pone.0287701 (PMC10370768; doi:10.1371/journal.pone.0287701)
Supplement: S2 Table — (DOCX) [file pone.0287701.s002.docx]

**S2 Table.** Percentage of arrests in school areas and arrest density in school areas pre- and post-remote learning periods by age group and race/ethnicity^[[1]](#footnote-1)^ (300-foot buffer)

|  | | | | **Weekly Arrest Density in School Areas (Arrests/km2)** | | | |
| --- | --- | --- | --- | --- | --- | --- | --- |
| **Age (Years)** | **Race/ Ethnicity** | **n Arrests** | **% Arrests in School Areas** | **Pre-Period Density** | **Remote Learning Period Density** | **% Change Remote v. Pre** | **Abs. Change Remote v. Pre** |
| **<18** | **Overall** | **16,434** | **11.3%** | **0.65 (0.62, 0.68)** | **0.27 (0.24, 0.3)** | **-58.6% (-63.9%, -53.0%)** | **-0.38 (-0.43, -0.34)** |
| <18 | Black | 10,448 | 10.3% | 0.38 (0.36, 0.4) | 0.14 (0.12, 0.17) | -62.4% (-68.6%, -54.9%) | -0.24 (-0.27, -0.2) |
| <18 | Hispanic | 4,566 | 14.0% | 0.22 (0.2, 0.24) | 0.11 (0.09, 0.13) | -50.1% (-60.6%, -40.5%) | -0.11 (-0.14, -0.08) |
| <18 | White | 793 | 9.0% | 0.03 (0.02, 0.03) | 0.01 (0, 0.01) | -65.2% (-84.1%, -38.4%) | -0.02 (-0.02, -0.01) |
| **18-24** | **Overall** | **69,974** | **8.7%** | **2.01 (1.97, 2.07)** | **1.16 (1.09, 1.22)** | **-42.6% (-46.0%, -39.1%)** | **-0.86 (-0.94, -0.78)** |
| 18-24 | Black | 36,668 | 7.6% | 0.93 (0.89, 0.96) | 0.51 (0.47, 0.55) | -44.5% (-49.5%, -39.9%) | -0.41 (-0.47, -0.36) |
| 18-24 | Hispanic | 22,895 | 11.1% | 0.84 (0.81, 0.88) | 0.49 (0.44, 0.53) | -42.4% (-48.1%, -35.9%) | -0.36 (-0.41, -0.29) |
| 18-24 | White | 6,304 | 5.6% | 0.12 (0.11, 0.13) | 0.06 (0.05, 0.08) | -48.5% (-61.2%, -32.1%) | -0.06 (-0.08, -0.04) |
| **25-44** | **Overall** | **186,274** | **8.5%** | **5.2 (5.11, 5.29)** | **3.11 (3.01, 3.21)** | **-40.3% (-42.5%, -37.8%)** | **-2.09 (-2.24, -1.94)** |
| 25-44 | Black | 87,345 | 7.7% | 2.21 (2.16, 2.27) | 1.26 (1.19, 1.32) | -43.2% (-46.4%, -39.7%) | -0.96 (-1.04, -0.87) |
| 25-44 | Hispanic | 60,349 | 11.1% | 2.16 (2.1, 2.21) | 1.39 (1.33, 1.47) | -35.3% (-38.9%, -31.5%) | -0.76 (-0.85, -0.67) |
| 25-44 | White | 27,772 | 5.7% | 0.52 (0.49, 0.55) | 0.3 (0.27, 0.34) | -42.1% (-48.1%, -33.6%) | -0.22 (-0.26, -0.17) |
| **45-64** | **Overall** | **69,352** | **8.0%** | **1.86 (1.81, 1.92)** | **1.01 (0.95, 1.07)** | **-45.6% (-49.2%, -41.9%)** | **-0.85 (-0.92, -0.77)** |
| 45-64 | Black | 34,148 | 7.4% | 0.84 (0.81, 0.88) | 0.46 (0.42, 0.5) | -45.3% (-50.8%, -39.6%) | -0.38 (-0.44, -0.32) |
| 45-64 | Hispanic | 18,937 | 10.6% | 0.67 (0.64, 0.7) | 0.37 (0.34, 0.41) | -44.1% (-49.7%, -38.2%) | -0.3 (-0.34, -0.25) |
| 45-64 | White | 11,905 | 6.0% | 0.24 (0.23, 0.26) | 0.12 (0.1, 0.14) | -52.7% (-61.0%, -42.1%) | -0.13 (-0.16, -0.1) |
| **65+** | **Overall** | **4,740** | **7.2%** | **0.12 (0.1, 0.13)** | **0.06 (0.05, 0.08)** | **-46.4% (-59.7%, -29.2%)** | **-0.05 (-0.07, -0.03)** |
| 65+ | Black | 1,965 | 7.4% | 0.05 (0.04, 0.05) | 0.03 (0.02, 0.04) | -31.1% (-52.9%, -1.6%) | -0.01 (-0.03, 0) |
| 65+ | Hispanic | 1,193 | 8.9% | 0.04 (0.03, 0.04) | 0.02 (0.01, 0.02) | -53.0% (-72.5%, -26.5%) | -0.02 (-0.03, -0.01) |
| 65+ | White | 1,183 | 5.8% | 0.03 (0.02, 0.03) | 0.01 (0, 0.01) | -75.3% (-92.0%, -49.0%) | -0.02 (-0.03, -0.01) |

1. Individuals classified as “other” race/ethnicity are included in the total/overall for each age category, but not shown separately. [↑](#footnote-ref-1)
