# Supplementary material for: School closures significantly reduced arrests of black and latinx urban youth
Source: PLoS One. 2023 Jul 26;18(7):e0287701. doi: 10.1371/journal.pone.0287701 (PMC10370768; doi:10.1371/journal.pone.0287701)
Supplement: S3 Table — (DOCX) [file pone.0287701.s003.docx]

**S3 Table.** Sensitivity analysis for percentage of arrests in school zones and arrest density in school areas pre- and post-remote learning periods by age group and race/ethnicity^[[1]](#footnote-1)^ using 1,000-foot and 2,640-foot buffer zones

|  | | | **Weekly Arrest Density in School Zones (Arrests/km2)** | | | |
| --- | --- | --- | --- | --- | --- | --- |
| **Race/ Ethnicity** | **n Arrests** | **% Arrests in School Zones** | **Pre-Period Density** | **Remote Learning Period Density** | **% Change Remote v. Pre** | **Abs. Change Remote v. Pre** |
| **[A] 1,000 Foot Buffer (NY & PA)** | | | | | | |
| **< 18 Years** | | | | | | |
| **Overall** | **16,434** | **65.7%** | **0.47 (0.46, 0.47)** | **0.21 (0.2, 0.22)** | **-54.2% (-56.5%, -51.8%)** | **-0.25 (-0.27, -0.24)** |
| *Black* | *10,448* | *64.4%* | *0.29 (0.29, 0.3)* | *0.13 (0.12, 0.14)* | *-55.2% (-57.7%, -52.3%)* | *-0.16 (-0.17, -0.15)* |
| *Hispanic* | *4,566* | *72.6%* | *0.14 (0.14, 0.15)* | *0.07 (0.07, 0.08)* | *-49.9% (-54.3%, -45.6%)* | *-0.07 (-0.08, -0.06)* |
| *White* | *793* | *50.3%* | *0.02 (0.02, 0.02)* | *0.01 (0.01, 0.01)* | *-62.0% (-71.7%, -51.1%)* | *-0.01 (-0.01, -0.01)* |
| **18-24 years** | | | | | | |
| **Overall** | **69,974** | **61.6%** | **1.78 (1.77, 1.8)** | **1.04 (1.02, 1.06)** | **-41.6% (-42.9%, -40.4%)** | **-0.74 (-0.77, -0.72)** |
| *Black* | *36,668* | *60.6%* | *0.92 (0.91, 0.93)* | *0.54 (0.53, 0.56)* | *-40.9% (-42.6%, -39.1%)* | *-0.38 (-0.39, -0.36)* |
| *Hispanic* | *22,895* | *68.8%* | *0.65 (0.64, 0.66)* | *0.38 (0.37, 0.4)* | *-41.4% (-43.5%, -39.0%)* | *-0.27 (-0.29, -0.25)* |
| *White* | *6,304* | *44.8%* | *0.12 (0.12, 0.12)* | *0.06 (0.05, 0.06)* | *-52.3% (-56.7%, -47.5%)* | *-0.06 (-0.07, -0.06)* |
| **25-44 years** | | | | | | |
| **Overall** | **186,274** | **61.6%** | **4.74 (4.72, 4.77)** | **2.79 (2.76, 2.82)** | **-41.2% (-42.0%, -40.4%)** | **-1.95 (-2, -1.91)** |
| *Black* | *87,345* | *62.2%* | *2.23 (2.21, 2.24)* | *1.36 (1.33, 1.38)* | *-39.1% (-40.3%, -37.8%)* | *-0.87 (-0.9, -0.84)* |
| *Hispanic* | *60,349* | *69.5%* | *1.75 (1.73, 1.76)* | *1 (0.98, 1.02)* | *-42.6% (-43.9%, -41.2%)* | *-0.74 (-0.77, -0.72)* |
| *White* | *27,772* | *45.3%* | *0.52 (0.51, 0.53)* | *0.3 (0.29, 0.31)* | *-42.0% (-44.4%, -39.4%)* | *-0.22 (-0.23, -0.2)* |
| **45-64 years** | | | | | | |
| **Overall** | **69,352** | **60.8%** | **1.78 (1.77, 1.79)** | **0.94 (0.92, 0.96)** | **-47.3% (-48.6%, -46.0%)** | **-0.84 (-0.87, -0.81)** |
| *Black* | *34,148* | *61.4%* | *0.88 (0.87, 0.89)* | *0.47 (0.46, 0.48)* | *-46.6% (-48.4%, -45.0%)* | *-0.41 (-0.43, -0.39)* |
| *Hispanic* | *18,937* | *70.0%* | *0.56 (0.55, 0.57)* | *0.3 (0.29, 0.31)* | *-47.0% (-49.0%, -44.7%)* | *-0.26 (-0.28, -0.25)* |
| *White* | *11,905* | *46.3%* | *0.23 (0.23, 0.24)* | *0.12 (0.11, 0.12)* | *-50.6% (-53.5%, -46.9%)* | *-0.12 (-0.13, -0.11)* |
| **65+ years** | | | | | | |
| **Overall** | **4,740** | **57.2%** | **0.12 (0.11, 0.12)** | **0.06 (0.05, 0.06)** | **-50.2% (-55.3%, -45.6%)** | **-0.06 (-0.07, -0.05)** |
| *Black* | *1,965* | *56.8%* | *0.05 (0.04, 0.05)* | *0.03 (0.02, 0.03)* | *-42.6% (-50.3%, -33.5%)* | *-0.02 (-0.02, -0.02)* |
| *Hispanic* | *1,193* | *71.5%* | *0.04 (0.03, 0.04)* | *0.02 (0.02, 0.02)* | *-48.6% (-57.1%, -39.7%)* | *-0.02 (-0.02, -0.01)* |
| *White* | *1,183* | *45.6%* | *0.02 (0.02, 0.03)* | *0.01 (0.01, 0.01)* | *-68.3% (-75.2%, -61.0%)* | *-0.02 (-0.02, -0.01)* |
| **[B] 2,640 Foot Buffer (SC)** | | | | | | |
| **< 18 Years** | | | | | | |
| **Overall** | **16,434** | **96.3%** | **0.25 (0.25, 0.26)** | **0.12 (0.11, 0.12)** | **-53.6% (-55.7%, -51.7%)** | **-0.14 (-0.14, -0.13)** |
| *Black* | *10,448* | *95.5%* | *0.16 (0.16, 0.16)* | *0.07 (0.07, 0.08)* | *-53.7% (-56.0%, -51.3%)* | *-0.09 (-0.09, -0.08)* |
| *Hispanic* | *4,566* | *99.1%* | *0.07 (0.07, 0.07)* | *0.04 (0.03, 0.04)* | *-50.6% (-54.3%, -46.7%)* | *-0.04 (-0.04, -0.03)* |
| *White* | *793* | *89.2%* | *0.01 (0.01, 0.01)* | *0 (0, 0.01)* | *-61.5% (-69.9%, -53.6%)* | *-0.01 (-0.01, -0.01)* |
| **18-24 years** | | | | | | |
| **Overall** | **69,974** | **95.4%** | **1.03 (1.02, 1.03)** | **0.59 (0.58, 0.59)** | **-42.8% (-43.9%, -41.8%)** | **-0.44 (-0.45, -0.43)** |
| *Black* | *36,668* | *94.6%* | *0.53 (0.53, 0.53)* | *0.31 (0.3, 0.32)* | *-41.5% (-42.7%, -39.9%)* | *-0.22 (-0.23, -0.21)* |
| *Hispanic* | *22,895* | *98.1%* | *0.35 (0.34, 0.35)* | *0.2 (0.19, 0.2)* | *-42.6% (-44.4%, -40.6%)* | *-0.15 (-0.15, -0.14)* |
| *White* | *6,304* | *89.0%* | *0.09 (0.09, 0.09)* | *0.04 (0.04, 0.05)* | *-50.9% (-54.2%, -47.4%)* | *-0.05 (-0.05, -0.04)* |
| **25-44 years** | | | | | | |
| **Overall** | **186,274** | **95.6%** | **2.73 (2.72, 2.74)** | **1.58 (1.56, 1.59)** | **-42.3% (-42.9%, -41.6%)** | **-1.15 (-1.18, -1.13)** |
| *Black* | *87,345* | *95.5%* | *1.27 (1.26, 1.27)* | *0.76 (0.75, 0.77)* | *-40.5% (-41.4%, -39.5%)* | *-0.51 (-0.53, -0.5)* |
| *Hispanic* | *60,349* | *98.4%* | *0.92 (0.91, 0.92)* | *0.52 (0.51, 0.53)* | *-43.1% (-44.2%, -41.9%)* | *-0.4 (-0.41, -0.38)* |
| *White* | *27,772* | *89.4%* | *0.38 (0.38, 0.38)* | *0.21 (0.21, 0.22)* | *-43.4% (-45.1%, -41.6%)* | *-0.16 (-0.17, -0.16)* |
| **45-64 years** | | | | | | |
| **Overall** | **69,352** | **96.1%** | **1.04 (1.04, 1.05)** | **0.54 (0.54, 0.55)** | **-47.9% (-48.9%, -46.9%)** | **-0.5 (-0.51, -0.49)** |
| *Black* | *34,148* | *96.5%* | *0.52 (0.51, 0.52)* | *0.27 (0.26, 0.27)* | *-47.8% (-49.2%, -46.3%)* | *-0.25 (-0.25, -0.24)* |
| *Hispanic* | *18,937* | *98.3%* | *0.29 (0.29, 0.3)* | *0.15 (0.15, 0.16)* | *-47.8% (-49.6%, -45.8%)* | *-0.14 (-0.15, -0.13)* |
| *White* | *11,905* | *91.0%* | *0.17 (0.17, 0.17)* | *0.09 (0.09, 0.09)* | *-47.4% (-49.4%, -44.7%)* | *-0.08 (-0.08, -0.07)* |
| **65+ years** | | | | | | |
| **Overall** | **4,740** | **94.4%** | **0.07 (0.07, 0.07)** | **0.03 (0.03, 0.04)** | **-52.7% (-56.4%, -48.7%)** | **-0.04 (-0.04, -0.03)** |
| *Black* | *1,965* | *93.5%* | *0.03 (0.03, 0.03)* | *0.02 (0.01, 0.02)* | *-46.3% (-52.4%, -39.7%)* | *-0.01 (-0.02, -0.01)* |
| *Hispanic* | *1,193* | *98.0%* | *0.02 (0.02, 0.02)* | *0.01 (0.01, 0.01)* | *-50.5% (-57.9%, -43.1%)* | *-0.01 (-0.01, -0.01)* |
| *White* | *1,183* | *90.7%* | *0.02 (0.02, 0.02)* | *0.01 (0.01, 0.01)* | *-64.7% (-70.0%, -58.8%)* | *-0.01 (-0.01, -0.01)* |

1. Individuals classified as “other” race/ethnicity are included in the total/overall for each age category, but not shown separately. [↑](#footnote-ref-1)
