# Supplementary material for: School closures significantly reduced arrests of black and latinx urban youth
Source: PLoS One. 2023 Jul 26;18(7):e0287701. doi: 10.1371/journal.pone.0287701 (PMC10370768; doi:10.1371/journal.pone.0287701)
Supplement: S4 Table — (DOCX) [file pone.0287701.s004.docx]

**S4 Table.** Percentage of city land area covered and percentage of youth and young adult^[[1]](#footnote-1)^arrests occurring in using buffer sizes of 300-ft (main analysis), 1,000-ft and 2,640-ft buffer zones, overall and by city

|  | | | | | **300-Foot Buffer (Boston)** | | | **1,000-Foot Buffer (NYC & Pittsburgh)** | | | **2,640-Foot Buffer (Charleston)** | | |
| --- | --- | --- | --- | --- | --- | --- | --- | --- | --- | --- | --- | --- | --- |
| **City** | **City Area (km2)** | **n Schools** | **n Youth Arrests** | **n Young Adult Arrests** | **% City Area** | **% Youth Arrests** | **% Young Adult Arrests** | **% City Area** | **% Youth Arrests** | **% Young Adult Arrests** | **% City Area** | **% Youth Arrests** | **% Young Adult Arrests** |
| **Overall** | **1,342.8** | **2,087** | **16,434** | **69,974** | **2.9%** | **11.3%** | **8.7%** | **23.1%** | **65.7%** | **61.6%** | **62.2%** | **96.3%** | **95.4%** |
| Boston, MA | 126.4 | 141 | 482 | 1,879 | 2.6% | 6.6% | 3.5% | 24.4% | 47.5% | 35.5% | 75.1% | 94.2% | 84.5% |
| Charleston, SC | 295.3 | 38 | 461 | 1,286 | 0.3% | 19.5% | 1.5% | 2.7% | 37.7% | 19.9% | 13.9% | 65.9% | 62.3% |
| New York City, NY | 778.0 | 1,839 | 14,585 | 63,310 | 4.2% | 11.5% | 9.4% | 32.6% | 69.2% | 65.7% | 78.9% | 99.2% | 98.2% |
| Pittsburgh, PA | 144.8 | 69 | 906 | 3,499 | 1.2% | 7.7% | 2.4% | 11.9% | 33.7% | 17.1% | 58.7% | 65.2% | 62.8% |

*Note:* See Figure S5 for maps showing the spatial extent of each buffer zone size.

1. Youth arrests are those among those aged <18 years, and young adult arrests are those occurring among individuals aged 18-24 years. [↑](#footnote-ref-1)
