# Supplementary material for: School closures significantly reduced arrests of black and latinx urban youth
Source: PLoS One. 2023 Jul 26;18(7):e0287701. doi: 10.1371/journal.pone.0287701 (PMC10370768; doi:10.1371/journal.pone.0287701)
Supplement: S1 Fig — (DOCX) [file pone.0287701.s005.docx]

Supporting Information

**S1 Fig.** Temporal Trends in Arrest Rates by Age Group in in Boston, Charleston, Pittsburgh, and New York City (2019-2020)


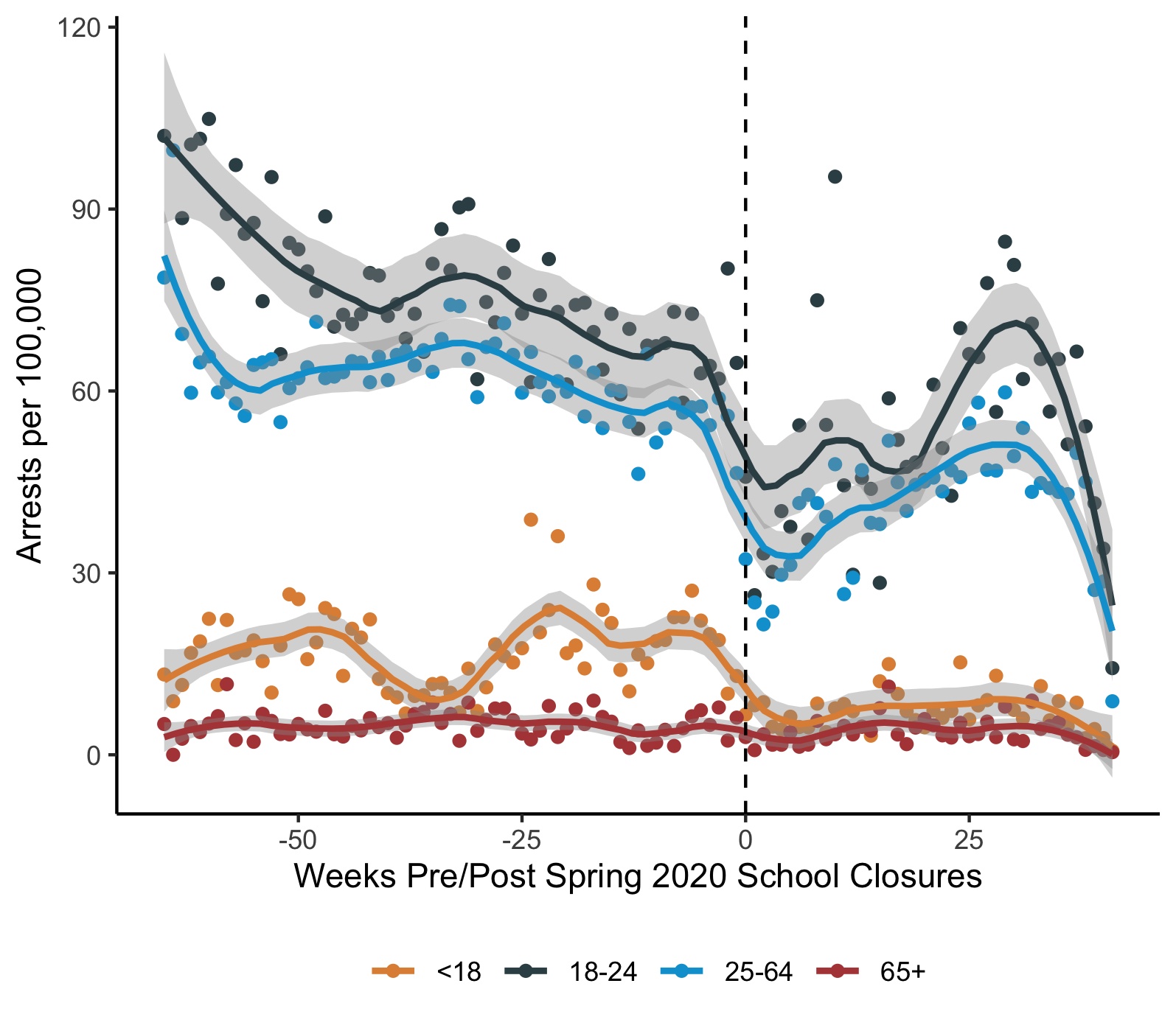


Note: Weekly averages smoothed using a loess function (span=0.25).
