# Supplementary material for: School closures significantly reduced arrests of black and latinx urban youth
Source: PLoS One. 2023 Jul 26;18(7):e0287701. doi: 10.1371/journal.pone.0287701 (PMC10370768; doi:10.1371/journal.pone.0287701)
Supplement: S2 Fig — (DOCX) [file pone.0287701.s006.docx]

**S2 Fig.** Change in Weekly Youth Arrests Rates After School Closures as Compared with Arrests During Summer 2019


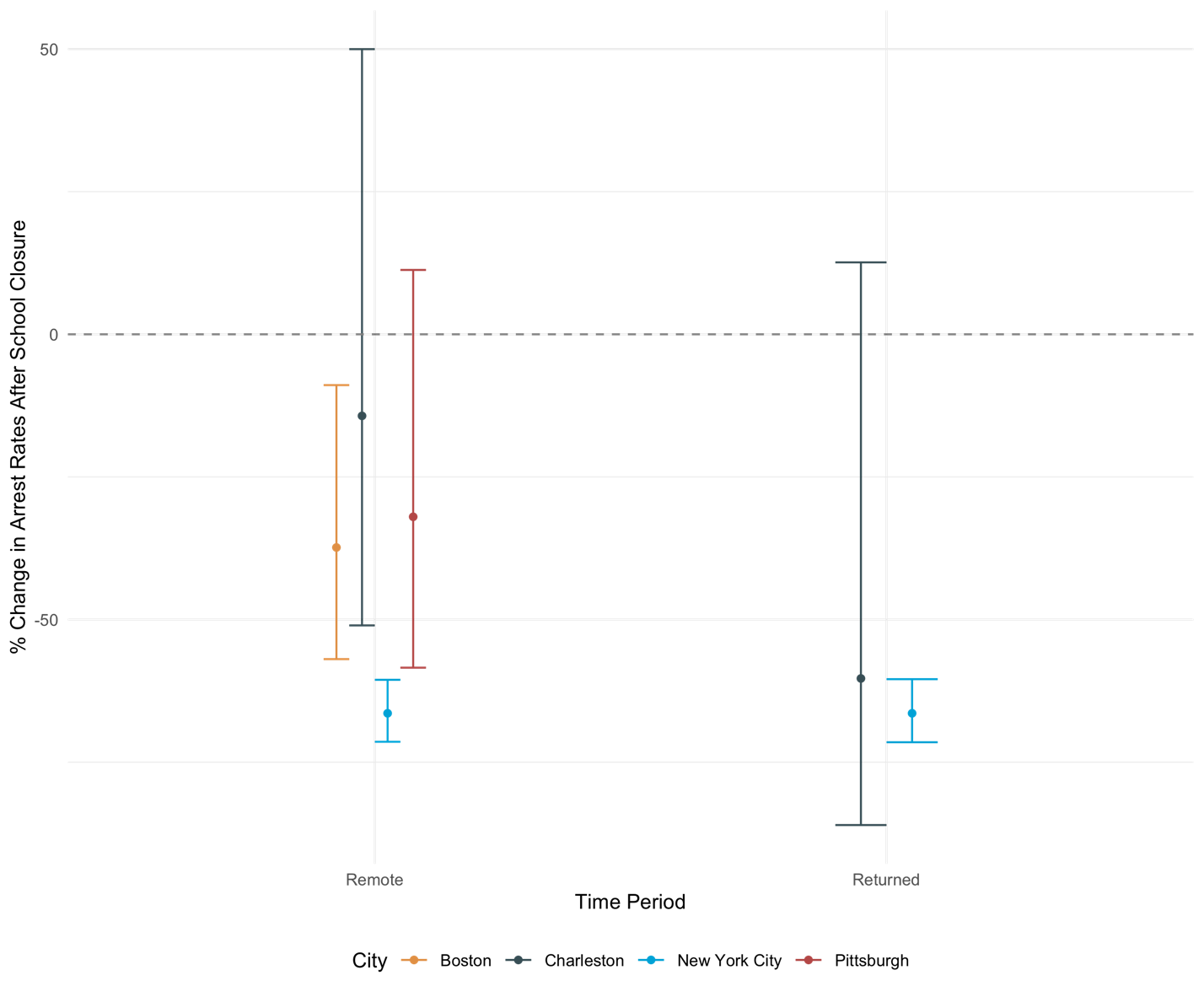


*Note*: Estimates from four city-specific interrupted time series negative binomial models predicting rates of arrests with a population offset and fixed effects for month. The referent time period for these models is June-August 2019. Only New York City and Charleston contribute data to the “Returned” period because Boston and Pittsburgh remained remote after September 2020. Full regression results available upon request.
