# Supplementary material for: School closures significantly reduced arrests of black and latinx urban youth
Source: PLoS One. 2023 Jul 26;18(7):e0287701. doi: 10.1371/journal.pone.0287701 (PMC10370768; doi:10.1371/journal.pone.0287701)
Supplement: S4 Fig — (DOCX) [file pone.0287701.s008.docx]

**S4 Fig.** Change in Weekly Youth Arrest Rates Before vs. After School Closures by Race and Ethnicity (2019-2020)

**
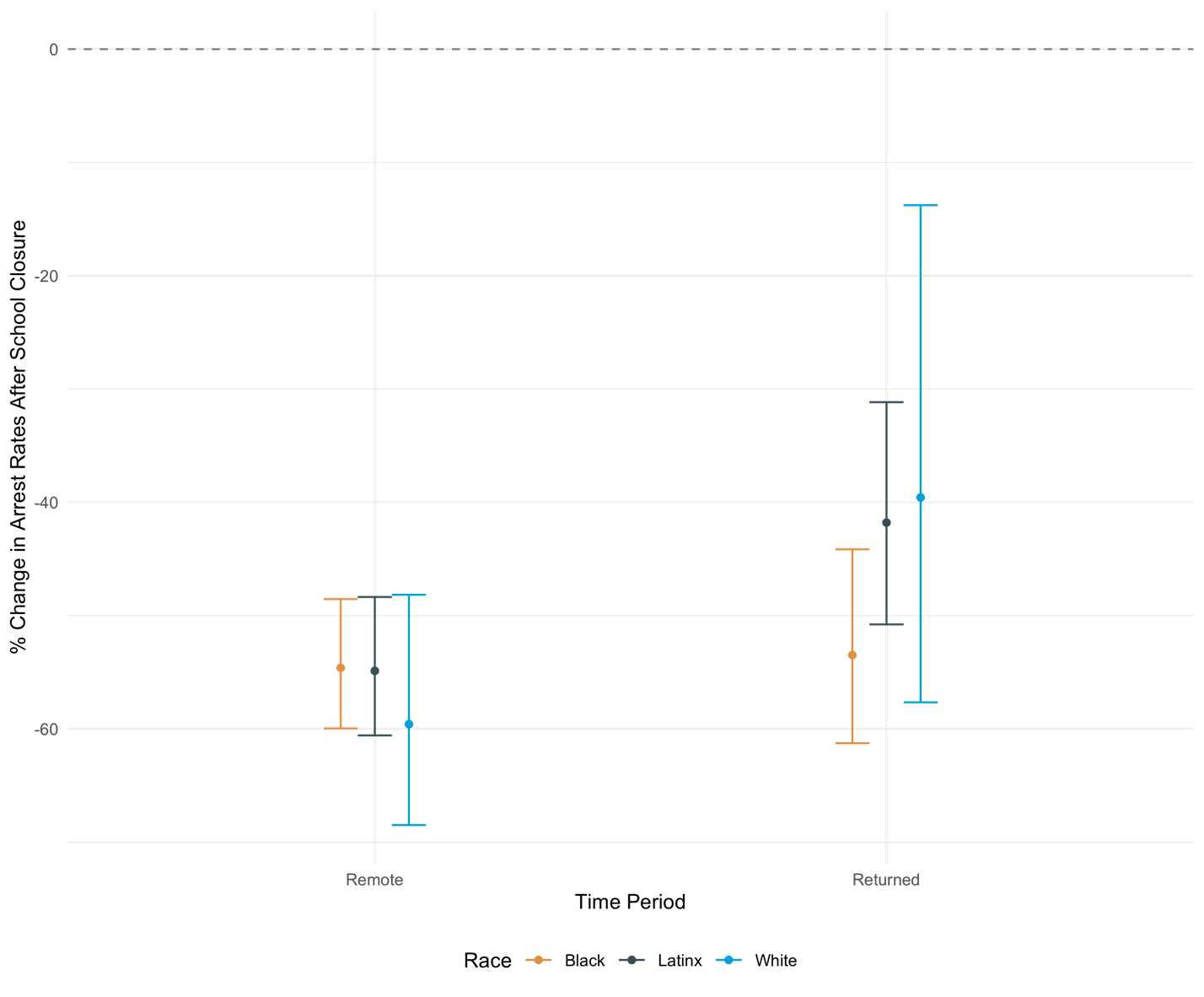
**

*Note*: Estimates from three race/ethnicity-specific interrupted time series negative binomial models predicting rates of arrests with a population offset and fixed effects for city and month. The referent time period for these models is 2019 to March 2020. Only New York City and Charleston contribute data to the “Returned” period because Boston and Pittsburgh remained remote after September 2020. Full regression results available upon request.
