# Supplementary material for: School closures significantly reduced arrests of black and latinx urban youth
Source: PLoS One. 2023 Jul 26;18(7):e0287701. doi: 10.1371/journal.pone.0287701 (PMC10370768; doi:10.1371/journal.pone.0287701)
Supplement: S5 Fig — (DOCX) [file pone.0287701.s009.docx]

**S5 Fig.** Spatial extent of different school buffer zone distances used in main (300-ft) and sensitivity analyses (1,000-ft and 2,640-ft)


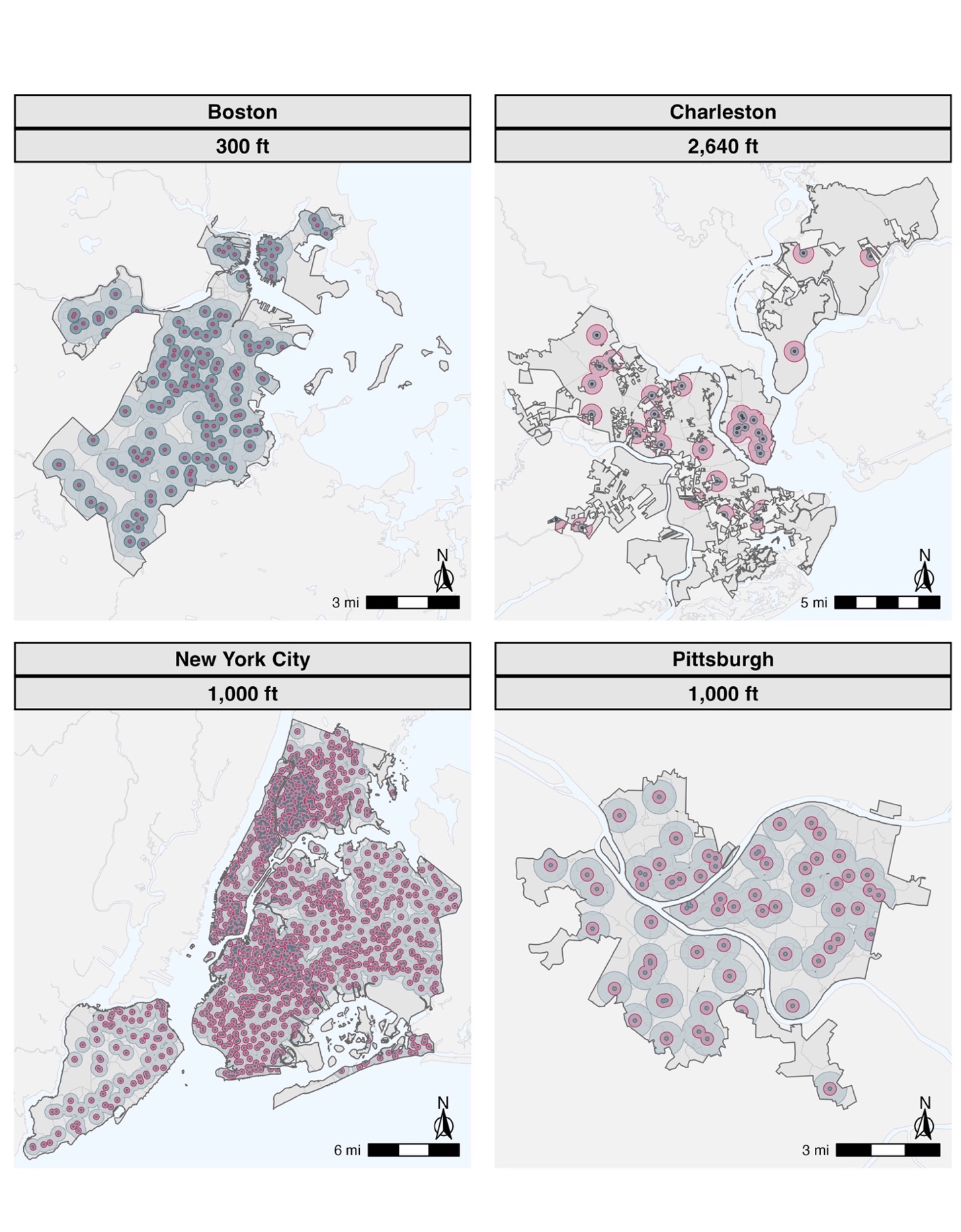


*Note:* For each city, the buffer distance used in states’ statute defining drug free zones is listed below the city name highlighted in red (Boston: 300-ft, Charleston: 2,640-feet, New York City: 1,000-ft, and Pittsburgh 1,000-ft). Each city is shown on a different spatial scale noted in the bottom right of each panel, however, the size of the buffer distances are equivalent across panels. For additional information about the amount of land area covered and the percentage of arrests occurring across different buffer sizes, see Table S4.
