# Supplementary material for: School closures significantly reduced arrests of black and latinx urban youth
Source: PLoS One. 2023 Jul 26;18(7):e0287701. doi: 10.1371/journal.pone.0287701 (PMC10370768; doi:10.1371/journal.pone.0287701)

**S8 Fig.** Weekly youth arrest density (arrests/km^2^) in school areas (300-foot buffer) and surrounding cities in pre- and post-remote learning periods, Pittsburgh, Pennsylvania


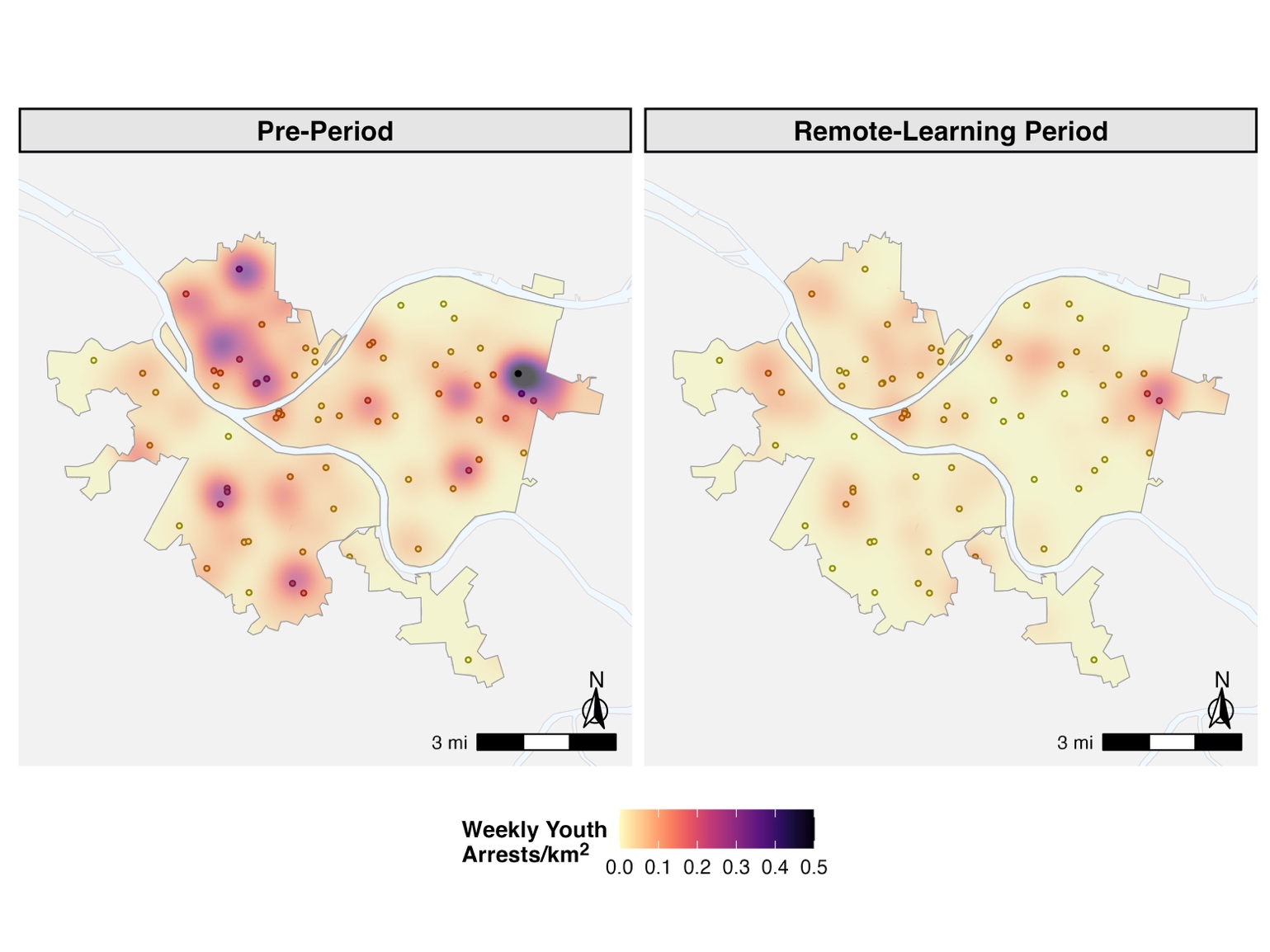

Supplement: S8 Fig — (DOCX) [file pone.0287701.s012.docx]
