# Supplementary material for: School closures significantly reduced arrests of black and latinx urban youth
Source: PLoS One. 2023 Jul 26;18(7):e0287701. doi: 10.1371/journal.pone.0287701 (PMC10370768; doi:10.1371/journal.pone.0287701)
Supplement: S9 Fig — (DOCX) [file pone.0287701.s013.docx]

**S9 Fig.** Percent change in arrest density for youth and young adult arrests within school buffer areas (300-feet) and outside of school buffer areas, overall and by race/ethnicity

**
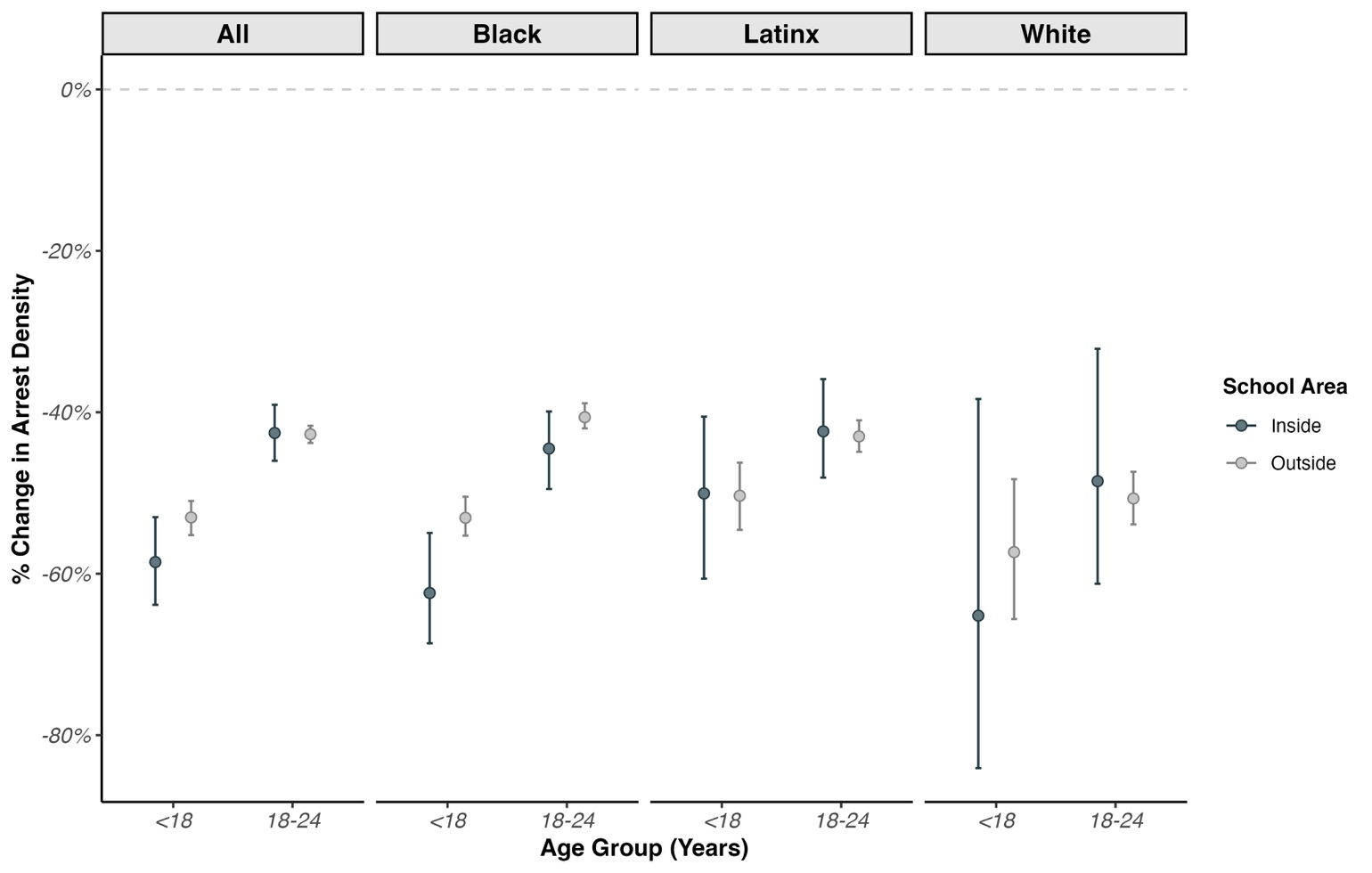
**
