# Supplementary material for: School closures significantly reduced arrests of black and latinx urban youth
Source: PLoS One. 2023 Jul 26;18(7):e0287701. doi: 10.1371/journal.pone.0287701 (PMC10370768; doi:10.1371/journal.pone.0287701)
Supplement: Attachment — Submitted filename: Review of PONE-D-22-31399.pdf [file pone.0287701.s015.pdf]

Review of PONE-D-22-31399

My overall take on your paper is that you are applying a clever research design by exploiting pandemic-era school closures as an identification strategy for analyzing the connection between (essentially) the social presence of potential (youth) offenders and those who arrest them. I think you can emphasize your research strategy somewhat more forcefully or prominently when motivating your paper and its attributes, especially in the introduction.

The introduction of the paper should speak more pointedly to the question of why, as scholars or as a society, we should care about "harm" to youths arrested for committing *actual* offenses? One could argue that they have *earned* their harm, and it may not be appropritate to expect society to care about that harm.....A passage on page 5 illustrates what I mean a little more clearly. There, you assert that schools embedded in neighborhoods create "criminal justice contact" that leads to criminalization of youth of color, thus affecting (presumably adversely) their "orientation towards both schooling and the criminal justice system." I can see how this would be a problem when youth of color are targeted by police for illegalities they did not commit or for the same (legal) activities undertaken by white kids, the theme of the example you cite later in that paragraph. But is there evidence that youth of color are not committing *actual* offenses (disproportionately)? The point is that at least some of the criminalization of youth of color that takes place in the context of neighborhood schools as policing hot spots might just be an artifact of race-criminality patterns that exist, independent of racially biased *policing*. Put another way, some youth (those who actually commit offenses) criminalize themselves by the choices they make; they are, after all, *youths*, and young people do make mistakes. (We all support actions that help them make better choices, both in general and in the aftermath of an arrest, but of course your paper is principally concerned with the actions of police, and that's fine.) Ultimately I think you could improve this passage, and thus the introduction overall, by being clearer on what the true problem actually is, and what it isn't, in the empirical setting you study here.

At the end of the short section on sensitivity analysis, you note, as part of your spatial analyses, that you considered school zone buffer distances of 1,000 feet for New York and Pennsylvania (it would be better if you said *Pittsburgh* here) and 2,640 feet for South Carolina (it would be better if you said *Charleston* here). I think this passage could be improved if you could give us a general feel for the context of these distances in practice and why you focus on two different distances for the two sets of cities. For example, within 1,000 feet of a typical (defined however you like) school in New York or Pittsburgh, is the landscape dominated by residences, or is it far enough away that you see no residences but instead a significant presence of stores or other buildings and establishments? Might there be a mix of these? Such detail would give the reader a clearer understanding of the lay of the land of the school neighborhoods that you're data cover.

Right now your paper is rather sparse on econometric details. When introducing your negative binomial regression model(s), you should show your regression equation in detail, making clear how your focus variable enters the equation and how its effect will be estimated, so that the reader understands the model's essential structure. It would also be helpful to remind the reader that you have count data, which motivates NB regression in the first place, and to comment on

why you are not estimating poisson models. Presumably, your data exhibit significant overdispersion that makes poisson regression invalid statistically, which can be readily tested; document and comment on the results of this test. Finally, accompanying these revisions, you would do well to show a table (or tables) showing the full results of your NB regression results, not just summaries of the *focus* results.

Although exploiting systematic school closures functions as an interesting identification strategy for your essential research question—helping you get at the effect of a policy-based, exogenous reduction in youth-police interaction in school neighborhoods—the closing of schools cannot be the lasting policy recommendation, as this is just not practical. Your paper needs to acknowlege this and emphasize that ultimately the lasting implication concerns the nature of *policing* in those neighborhoods.

Within the Discussion section, you note that a number of school districts have recently ended the use of school resource officers, which then draws attention to the role of police officers. I suspect many readers do not know this. You might find it interesting to tap into a recent study of mine, titled “Self-Protection Against Crime: What Do Schools Do?” (*Applied Economics* 2018). It’s possible that its findings, based on an economic analysis of guard use inside schools, could lend perspective to your closing discussion with respect to how you think about the problem you are studying and its empirical setting. Please note, however, that this is just a suggestion and by no means a requirement for your revision.
