# Supplementary material for: School closures significantly reduced arrests of black and latinx urban youth
Source: PLoS One. 2023 Jul 26;18(7):e0287701. doi: 10.1371/journal.pone.0287701 (PMC10370768; doi:10.1371/journal.pone.0287701)
Supplement: Attachment — Submitted filename: Reviewer Comments.pdf [file pone.0287701.s014.pdf]

Referee Comments (PONE-D-22-31399)

The study capitalizes on a natural experiment resulting from school shutdowns due to the COVID-19 pandemic to measure the impact of policing schools as "hot spots" on racial disparities of youth criminalization. Results are clear and compelling and have important implications for policy. I offer the following comments to potentially improve the manuscript.

Methods:

Multiple comparisons are made to support the claim that areas around schools are particularly important for the criminalization of Black and Latinx youth—comparisons between pre- and post-school closures, comparisons by race, comparisons by city, and comparisons by age group. Why not also compare arrest density of school buffers zones with arrest density outside of school zones? It would seem to be the most direct comparison to support the overall conclusion.

Also, the fixed effects approach is effective for controlling for unmeasured covariates that differ between cities (and season). What remains unexamined here are the potential confounders (or moderators!) due to within-city inequality based on variables that have been tested extensively in criminological research such as social disorganization, resource disadvantage, median income, percent HS grads, % aged 15-24, population density, etc. Indeed, Percent Black and Percent Latinx might even be most relevant here. That said, I think it's reasonable to argue that an extensive analysis is beyond the current scope, but a supplemental analysis to check for variation, say between school sites within cities, would be a helpful clarification (and/or signpost for future research).

Please clarify: Do arrest rates within the buffer zones include in-school arrests? How does this impact findings?

Results:

The text on pg. 12 (Youth Arrest Rates and Disparities…) combined with Figure 1 is unclear. Is the cited rate of average weekly youth arrest rates of 17.4/100,000 for 2019 meant to represent the average for the whole year? Does that include Jan-Mar 2020? Or does it represent only Jan-Mar 2020 rather than 2019? Also, perhaps I am not understanding what the trendlines represent, but why does the trajectory at the end of 2019 not line up with the beginning of 2020? Most importantly—from Jan-Mar 2020, the trajectory already appears to be heading downward. What could explain it? How do we rule out that the levels after Mar 2020 are not simply a continuation of processes that started in Jan, rather than the school shutdowns?

Also – please define IRR at some point in the study.

Very minor:

Pg. 7, line 106: I appreciate the magnitude of the impact of these findings on health outcomes, but I actually think the authors might be selling their reach here a bit short, as the findings also

have implications for economic outcomes (e.g., Pager 2003 – The Mark of Criminal Record), not to mention education, and future criminal justice contact as well.
